# Use of mixed-type data clustering algorithm for characterizing temporal and spatial distribution of biosecurity border detections of terrestrial non-indigenous species

**DOI:** 10.1371/journal.pone.0272413

**Published:** 2022-08-09

**Authors:** Barbara Kachigunda, Kerrie Mengersen, Devindri I. Perera, Grey T. Coupland, Johann van der Merwe, Simon McKirdy

**Affiliations:** 1 Harry Butler Institute, Murdoch University, Murdoch, WA, Australia; 2 School of Mathematical Sciences, Queensland University of Technology, Brisbane, QLD, Australia; 3 Faculty of Computing and Technology, University of Kelaniya, Colombo, Sri Lanka; Zoological Survey of India, INDIA

## Abstract

Appropriate inspection protocols and mitigation strategies are a critical component of effective biosecurity measures, enabling implementation of sound management decisions. Statistical models to analyze biosecurity surveillance data are integral to this decision-making process. Our research focuses on analyzing border interception biosecurity data collected from a Class A Nature Reserve, Barrow Island, in Western Australia and the associated covariates describing both spatial and temporal interception patterns. A clustering analysis approach was adopted using a generalization of the popular k-means algorithm appropriate for mixed-type data. The analysis approach compared the efficiency of clustering using only the numerical data, then subsequently including covariates to the clustering. Based on numerical data only, three clusters gave an acceptable fit and provided information about the underlying data characteristics. Incorporation of covariates into the model suggested four distinct clusters dominated by physical location and type of detection. Clustering increases interpretability of complex models and is useful in data mining to highlight patterns to describe underlying processes in biosecurity and other research areas. Availability of more relevant data would greatly improve the model. Based on outcomes from our research we recommend broader use of cluster models in biosecurity data, with testing of these models on more datasets to validate the model choice and identify important explanatory variables.

## Introduction

Preventing non-indigenous species (NIS) establishing in new locations is key to effective biosecurity. Strategies to prevent establishment include prevention of NIS arrival, early detection, and eradication [1]. Preventing NIS arriving, followed by early detection, are the most effective forms of biosecurity [2], as once NIS are established, eradication is problematic [3]. Adverse consequences of invasions vary from the mere presence of NIS in areas where they have not previously been detected, that are deemed undesirable and detrimental, to the destruction of entire ecosystems [4]. As such, it is imperative that NIS are intercepted before they become established. Stringent border detection contributes significantly to this outcome [5].

Developing robust inspection protocols and surveillance strategies are critical components of plant and animal biosecurity measures. Optimizing the use of all available biosecurity detection data, even when these data are incomplete, coupled with detailed analyses, will enhance the capacity to make effective biosecurity decisions. If biosecurity measures are sufficiently competent, there is often a large proportion of zero values for detections, a good indicator that materials brought in are complying with sanitary and phytosanitary measures [6]. Generally, biosecurity risk material and pests occur at low densities during biosecurity border inspections, making biosecurity surveillance inevitably imperfect [7]. When organisms are detected, values may range from a single organism/unit to extreme values depending on the type and origin of commodity inspected, pre-border and in-transit quarantine protocols, and the introductory pathways [8, 9]. The very nature of this data, with large proportions of zeros, a mixture of distributions and the count nature of data can make statistical analyses of biosecurity data challenging.

Biosecurity border inspections are important to any biosecurity management programme in that: 1) interception data provides information about risks associated with individual or groups of NIS species pests to inform biosecurity actions; 2) inspections provide information about risks associated with specific commodities and introduction pathways; 3) inspections monitor and evaluate the effectiveness of phytosanitary treatments and pre-border biosecurity protocols and: 4) in wider context, border inspections identify problematic importing agents for refusal of entry of consignment, destruction or fines, or for targeting with more rigorous inspections [10, 11].

Probability of border interception changes with inspection effort relative to the volume and type of cargo, the introductory pathway, and the biological characteristics of the NIS that influence detection e.g. growth stage [12]. Border interception rate is a useful proxy for arrival rate for individual species, and was previously thought to be correlated with establishment probability [13]. However, it has since been established that the relationship between interception and establishment is weak, and interception rates are poor predictors of an incursion [14].

Biosecurity border inspection data are rarely made available other than to designated organizations and departments [5]. Data inaccessibility is primarily due to the complexity of the databases, sensitivity of the information, confidentiality and privacy concerns, and the potential for misinterpretation and misuse of information, as evidenced in trade disputes [15]. This lack of data availability makes statistical analysis and interpretation of biosecurity data problematic, limiting the scope for statisticians to explore underlying patterns in biosecurity data.

A common aim when analyzing any data set is to choose an appropriate statistical model from a set of candidates. Fitting an appropriate statistical model is crucial for correct data interpretation. The choice of fitted distributions is dictated by either the stochastic process governing the outcome of interest or by observing its empirical distribution [16]. Common statistical approaches include normal linear regression with a log-transformed response and generalized linear models (GLM) with Poisson or negative binomial distributions for the response [17–19].

With biosecurity data, overdispersion is a problem as data are comprised of counts, and invalid inferences may occur if overdispersion is not addressed. Overdispersion may result from population heterogeneity, misspecification of the model, omission of important covariates, presence of outliers, non-independence of data, and a high proportion of zero events in relation to the Poisson distribution [20–22]. The negative binomial distribution allows for some forms of over-dispersion, notably caused by rare events, and improves on the use of the standard Poisson [23]. Using zero-inflated or hurdle models is a common approach to deal with an excess number of zero counts as well as over-dispersion [24]. Both models can reduce bias from extreme non-normality of the data and can provide more accurate estimates of model coefficients than the standard models [25]. As such, these models are better suited than others to cope with the idiosyncrasies associated with biosecurity data.

A more general approach to analysis of biosecurity data is to consider that the data are a mixture of subgroups. The composition of these clusters can then be examined to provide new insights into the distribution of the data and the underlying system or process [26]. Clustering is unsupervised classification where data are classified without the knowledge of the class labels and provides intuitive interpretation of the relevant aspects of the data at hand [27]. A clustering approach can be used to describe diverse forms of over-dispersion and population heterogeneity where the distribution might be multimodal, skewed, or non-standard [28, 29]. For example, in the context of this case study, clusters can be constructed to describe the large proportions of zero and single counts, as well as relatively large values. Clusters can also provide more insight into characterization of the cluster components with respect to environmental, geographic, and other covariates.

Biosecurity data used in this study were collected as part of industrial development on a remote island (Barrow Island, Australia) and analyzed to inform biosecurity management decisions. The industrial project on the island was permitted with the proviso that no new NIS be introduced to the island [30, 31]. Non-indigenous species (NIS), referred to also as non-native, alien, or exotic organisms) are species that have been introduced outside of their natural previous or present range by human activities and if established, can threaten the local biodiversity or ecosystems [32]. Invasive alien species are those introduced to a novel environment with negative ecological, economic, or social impacts [33]. The current biosecurity surveillance monitoring programme on Barrow Island is monitoring all NIS, inclusive of invertebrates, vertebrates, plants, and marine species except microorganisms. There are a range of NIS species that have been classified as high-risk to the island and are on a priority watch list across the biosecurity continuum as their establishment on the island have undoubtably devastating impacts on the natural ecosystem of Barrow Island [34]. High risk species were identified from a suite of species based on their potential to be introduced, the difficulty of detecting the species, and the amount of damage they were perceived to cause should they establish [34, 35]. Species were also identified based on their known invasiveness elsewhere in the world, for example the highly invasive species *Rattus rattus* (black rat), *Cenchrus ciliaris* (buffel grass), *Hemidactylus frenatus* (Asian House Gecko), *Monomorium destructor* (Singapore ant) and *Pheidole megacephala* (big-headed ant) were prominent on the surveillance radar [36, 37]. It should be highlighted that for Barrow Island, all NIS were unacceptable, and detection was mandatory [34, 38, 39]. There are 22 confirmed or putative non-indigenous invertebrate species recorded on Barrow Island and no established vertebrate non-indigenous species [40, 41].

To help achieve this, biosecurity surveillance and management has been conducted on Barrow Island since 2009 and will continue for the life of the project. Biosecurity surveillance data are used to assess the success of the various aspects of an on-going environmental program complemented by pre-border inspection protocols, a border clearance program, and post-border biosecurity surveillance program [30]. All NIS species that have been detected on Barrow Island have been eradicated, e.g. the Asian House Gecko in 2015, or are under a quarantine response like the buffel grass [42]. To date, on-going NIS species surveillance has not detected the presence of these species [5].

For our study, we used border inspection data collected on Barrow Island between 2009 and 2015 during the construction phase of the liquefied natural gas (LNG) plant on the island. The data were used to assess the contribution of type of detection, phase of project, season, and physical inspection location on the island in characterizing biosecurity border detection events.

The motivation to conduct this study was to find a more effective way of assessing biosecurity data, data that are often complex, skewed by a large proportion of zeros, a mixture of distributions and has a count nature. All these factors make statistical analysis problematic, often violating assumptions of common statistical tests. The aim of this paper is to assess the performance of a clustering approach to characterizing biosecurity interception data in terms of its capacity to manage these difficulties and assess the data both temporally and spatially. Implementing Huang’s k-prototypes algorithm for mixed-type data, we explore the clustering approach using a specific set of explanatory variables collected as part of border interception biosecurity data for terrestrial NIS collected at Barrow Island [43, 44]. Two complementary analyses approaches were used, without and with covariates included in the model. This paper follows preliminary analyses conducted on invasive terrestrial species on Barrow Island by Scott, 2017 [5]. The overarching goal of the research is to improve biosecurity management protocols and strategies to minimize the introduction of NIS in a global context.

## Material and methods

### Study site

**Fig 1:**
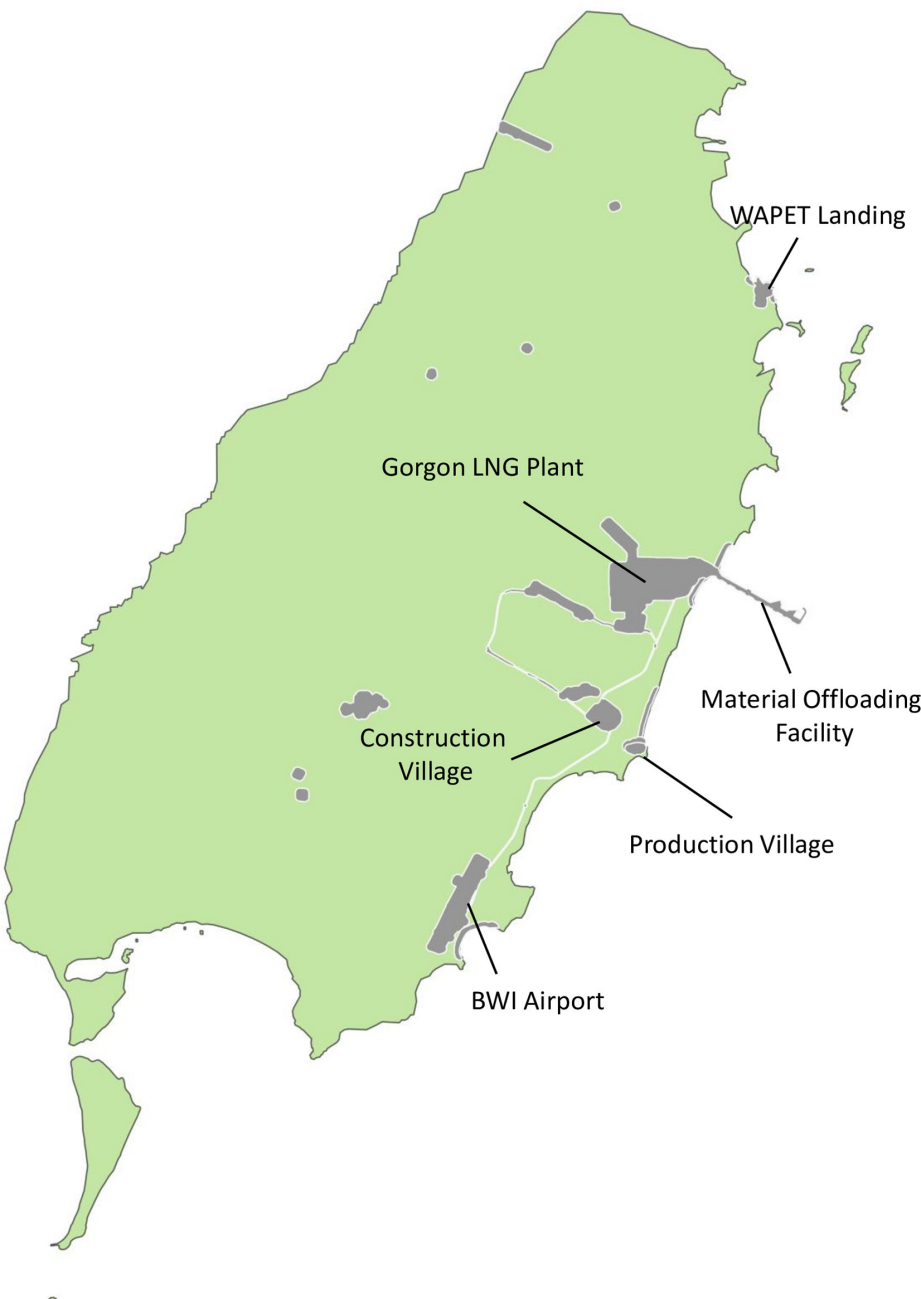
Physical definitions of biosecurity borders inspection points on Barrow Island, Australia. The map is an indicative representation of the biosecurity inspection sites between 2009 and 2015. Map created using the Free and Open Source QGIS. https://qgis.org/en/site/ [46]. The shapefiles were provided by Chevron Australia Pty Ltd. This work has been licensed under CC BY 4.0.

Barrow Island (BWI) is located at 200 45´S, 115025´E, and 56 km off the mainland of Australia. It is 25 km long, 10 km wide, covering an area of approximately 23 400 hectares above the high-tide mark [31] (Fig 1). The Gorgon Liquefied Natural Gas Project (LNG) plant is situated on BWI and was developed to process extensive gas resources from the Gorgon and Jansz-Io gas fields in the North-West Basin, Australia. The Gorgon LNG Plant occupies 300 ha, about 1.3% of the Barrow Island land area.

#### Biosecurity system and border biosecurity inspection protocols

The Quarantine Management System (QMS) was developed by Chevron and implemented to protect the conservation values of Barrow Island [5, 37]. The overarching aim of the QMS was to prevent the introduction and establishment of vertebrate, invertebrate, and plant NIS [45]. The biosecurity protocols implemented were specific to identified risks and the implementation of safeguards for the diverse types of cargo and within the logistic chain, e.g., site management, quarantine management plans for contractors and vendors. Types of biosecurity risk material were categorized into five groups: vertebrates, invertebrates, seed, soil/organic matter, and other plant material.

Border inspection methods included visual, manual, and physical inspection, detector dogs, and x-ray technology [47]. A cabinet x-ray was used for passenger screening at airports [35]. A colour-coded tagging system and inspection process was implemented to ensure compliance with all biosecurity management activities. This ensured that one hundred percent of cargo transported to Barrow Island was inspected [5].

### Statistical methodology

#### Data collection

The dataset used for this study was collected during the construction phase on Barrow Island, between 2009 and 2015. The unit of inspection, termed the Material Management Ticket (MMT), was used for biosecurity inspection on the island. The MMT is a system used to track the movement of materials and can include one or several containers, or groupings of similar materials for biosecurity inspection [5]. An MMT can also encompass one or more inspections. When no NIS was found during cargo inspection, results of inspection were recorded as zero. Otherwise, a detailed set of records was recorded for positive detections. A detection was either a specimen of an organism or multiple specimens of an organism [5]. A unit was an individual count of the type of material detected.

When an MMT was identified as positive, appropriate remedial treatment action was applied, e.g., fumigation or the item was refused entry. Cargo were re-inspected at sites where the goods were to be used and can be considered as a quality assurance measure to ensure that the cargo reaching the island was free of NIS [5].

The following variables were identified as covariates to be used in the analysis, namely; 1. physical location (biosecurity border inspection entry point), 2. type of detection, 3. construction phase of the project, and 4. season.

### 1. Physical location (biosecurity border inspection entry point)

In biosecurity, a border is the point of entry of goods by land, air, or sea into a designated area for quarantine purposes [48]. Border inspection points at BWI are identified as Barrow Island Airport, BWI Material Offloading Facilities (MOF), and Western Australian Petroleum Landing Site Landing (WAPET Landing) (Fig 1). Secondary border inspection occurred after final cargo clearance and included points where the consignments are offloaded for use on BWI, such as the Gorgon LNG Plant, the Construction Village, the Production Village, and Western Australia (WA) Oil Camp. Border detection was primarily through biosecurity surveillance and secondly by citizen science, since all personnel coming to work on the island are trained and obligated to report any suspected NIS plant or animal species [42].

### 2. Type of detection

These were classified as vertebrates, invertebrates, seeds, and plant materials. Vertebrates included mammals, birds, reptiles (snakes and geckos) and amphibians. Invertebrates included arthropods, termites, worms, ants, and terrestrial molluscs. Plant materials included twigs, leaves, grass, roots, and remnants of fruits. All the detection events were recorded and classified according to their impact on biodiversity on BWI. The detections were subsequently and taxonomically classified to genus level depending on the condition of the specimen.

### 3. Construction phase of the project

Construction activities were described as phases: early construction (2009–2011), main construction phase (2012–2014), and transition phase (2015). Early construction phase consisted mostly of site preparations and earthworks; while main construction phase consisted mainly of major construction activities relating to the building of the three liquefied natural gas (LNG) processing plants and all the supporting infrastructure, such as gas turbine generators, slug catchers, Boil Off Gas (BOG) flare, MR/PR compressors. Finally, the transitional phase consisted of preparations for start-up, commissioning tests and eventual start-up and initial operations [49].

### 4. Season

Data were classified into four seasons representing the time periods: January-March (autumn—1), April-June (winter—2), July-September (spring—3) and October-December (summer—4). Barrow Island is arid and has a subtropical climate. Summer and autumn are characterised by high temperatures (20–34°C) with high humidity while winter is characterised by moderate temperatures (17–26°C) with fine weather [50]. Annual average rainfall is 320 mm with cyclonic events bringing 30mm—300 mm of rainfall in one cyclonic event [51].

As such, the amount of cargo, type of cargo and personnel reaching biosecurity borders on the island varied significantly as the project progressed, directly impacting the number of biosecurity detections. Table 1 gives a summary of the covariates which were considered for the clustering algorithm.

**Table 1:** Description of covariates used in the cluster model based on biosecurity detections from Barrow Island from 2009 to 2015.

Covariate	Levels	Factor levels
Physical location (biosecurity border inspection entry point)	8	1. Barrow Island Airport (BAirport) 2. Barge -floating accommodation 3. Construction Village (ConstV) 4. Production Village (ProdV) 5. WAPET Landing 6. Materia Offloading Facility (MOF) 7. LNG Plant (LNGP) 8. Others
Type of detection	4	1. Invertebrates 2. Seeds 3. Vertebrates 4. Plant material
Phase	3	1. Early construction (2009–2011) 2. Major construction (2012–2014 3. Transition (2015)
Season	4	1. January-March (autumn—1) 2. April -June (winter– 2) 3. July–September (spring– 3) 4. October -December (summer– 4)

N.B: Other includes Permanent Operating Facility (POF), Gas Treatment Plant (GTP), Quarantine Approved Premises (QAP).

#### Clustering

Distance-based algorithms, such as k-means, are very popular due to their simplicity, interpretability, and ease of implementation [52, 53]. Further, statistically desirable characteristics of the clusters include the stability of identified clusters, independence of variables within a cluster, and the degree to which a cluster can be well-represented by its centroid in mixed data type [54]. A popular approach that allows for mixed-type data is Huang’s k-prototypes algorithm [43, 44], which calculates the distances between objects and cluster centroids for categorical and continuous variables, and combines them in a single objective function [44]. For k-prototypes, cluster centres are represented by mean values for numeric features and mode values for categorical features.

Two clustering analyses were considered here. The first involved clustering only the response count data and the second included both the response variable and covariates.

For the first cluster analysis, many available software packages analyze univariate continuous data [55, 56]. Here, the Ckmeans.1d.dp algorithm [57] was implemented using Ckmeans.1d.dp in R software, to determine the clusters using univariate log-transformed detection counts. The algorithm guarantees the optimality of clustering by ensuring that the total of within-cluster sums of squares is always the minimum given the number of clusters k.

For the second analysis, several software packages in R are available for clustering mixed-type data (clustMixType [58], clustMD [59], Gower’s similarity matrix [60], ClustOfVar [61] and CluMix [62]). Here, the clustMixType package in R [58] based on Huang’s k-prototype algorithm [44] was used to assess the role of covariates: seasons, construction phases, and physical locations/sites where the biosecurity inspection was done. This package allows using combination of both numeric and categorical data in model fitting. The k-prototypes algorithm used belongs to the family of partitional cluster algorithms [63].

The steps of the algorithm were:

- Select k initial prototypes for k clusters from the date set X
- For each observation:
  - assign observations to its closest prototype according to d ().
  - update cluster prototypes by cluster-specific means/modes for all variables.
- Repeat Step 2 until no data object has changed clusters after a full cycle test of X.

Clusters are assigned using: [58]

$$d\left(x,\;y\right)=d\_\left(euclid\right)\;\left(x,\;y\right)+\lambda \;d\_\;\left\{simple,\;matching\right\}\;\left(x,y\right)$$ Eq 1

For numeric explanatory variables, results are given as summary statistics for each cluster, while for categorical variables, the results are as a proportion of the contribution of each factor level across each cluster in a tabular form. Further, summary profile histograms of the explanatory variables are given as well. The clusters are mutually exclusive.

The covariates represent the temporal aspect of the data since the data were collected over a period (2009–2015) where seasonality and other construction activities have a direct impact on the number and type of units detected.

An alternative partitioning method widely used, though not addressed in this paper, are finite mixture models, in which each cluster is assumed to follow some parametric distribution, the parameters of which are then typically estimated using the EM (expectation-maximization) algorithm [26, 64–66]. The data collected for this study were number of units detected (counts), the data is non-parametric. K-clustering which uses “hard” assignment, with the probability distribution of the data is unknown while the Expectation-Maximization (EM) algorithm uses “soft” assignment mechanism and each data point is assigned to every cluster centre according to its probability of generating the data thus optimizing the marginal likelihood of the data using a defined probability distribution, usually the Gaussian [67]. More recently, the work done by Behzadi, Müller [68] using ClicoT **(Cl**ustering mixed type data **I**ncluding **CO**ncept **T**rees), though not on biosecurity data might be an alternative approach to clustering mixed type data and complement other techniques for data classification.

## Results

### Data description

In total, over 600,000 inspections were conducted during the period December 2009 to December 2015, with only 5,380 biosecurity risk material detections, which translates to approximately 1% of the inspections. For this study, soil/organic matter data were excluded as it was privy to a different type of biosecurity assessment and analyses, hence the final sample size used was 5,325 units (Table 2).

**Table 2:** Summary statistics for the Barrow Island biosecurity detection events between 2009 and 2015.

Type of detection	Proportion	N	Mean	Median	min	max	std dev	Variance
**Invertebrate**	0.62	3325	3.61	1	1	1000	28.1	790.0
**Plant material**	0.09	469	2.41	1	1	100	5.5	29.7
**Seed**	0.26	1392	5.82	1	1	1000	33.7	1133.0
**Vertebrate**	0.03	139	1.58	1	1	20	2.3	5.3
**Grand Total**	**1.00**	**5325**	**4.03**	**1**	**1**	**1000**	**28.2**	**793.2**

**Fig 2:**
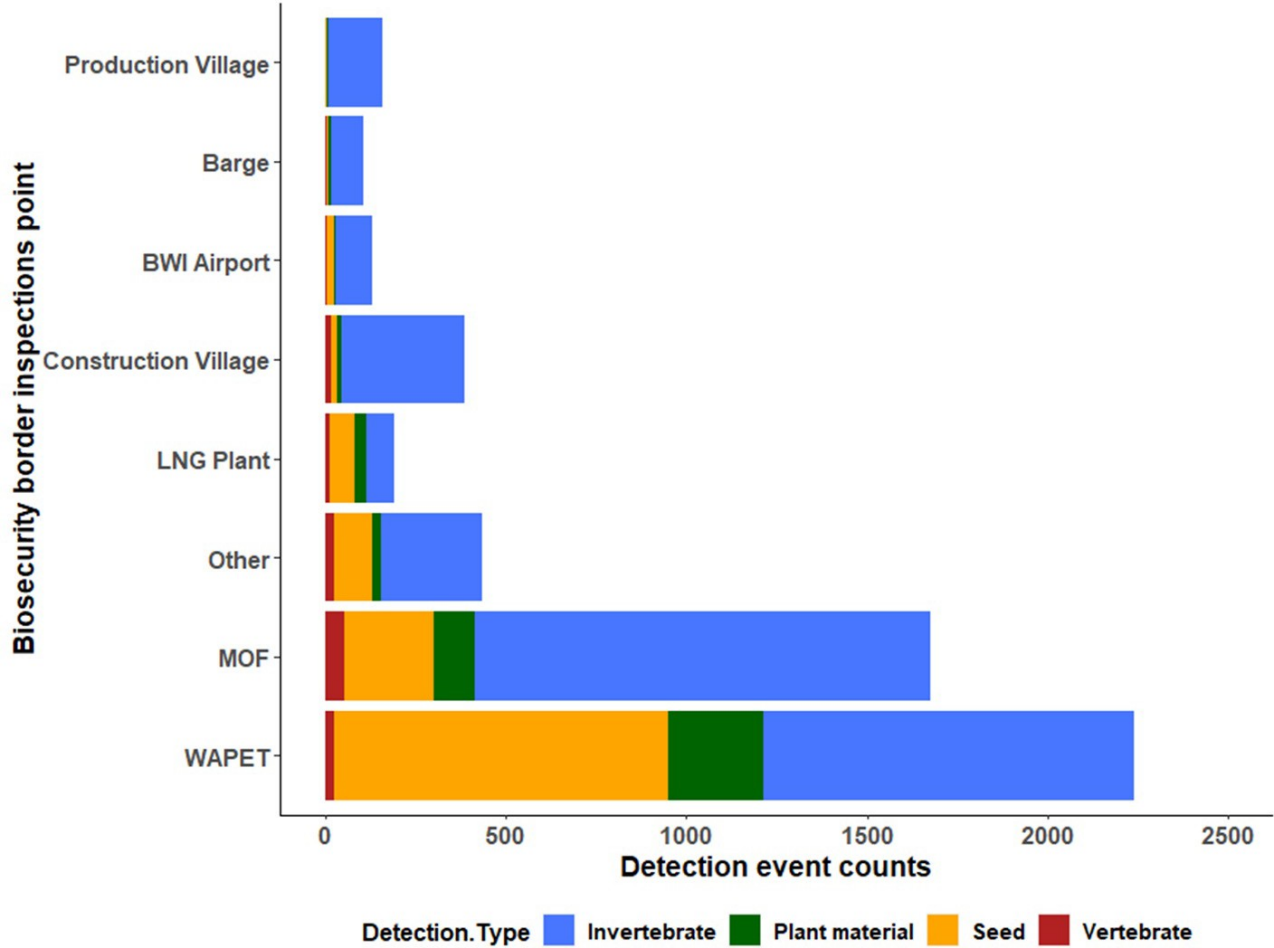
Comparison of biosecurity border inspection detections by physical border entry location for Barrow Island from 2009 to 2015. NB: Others included Barrow Island shore waters and pre-commissioning sites.

A high percentage (73%) of the border detection were at the primary biosecurity entry points, namely, the WAPET Landing and the Material Offloading Facility, contributing 42% and 31% respectively. The bulk of the construction material/consignments were received at these points. At final clearance inspection, detections constituted the remaining 27% of the detections (Fig 2), which were found at human-inhabited areas associated with food and perishables and at construction sites where high volumes of imported construction materials were delivered.

**Fig 3:**
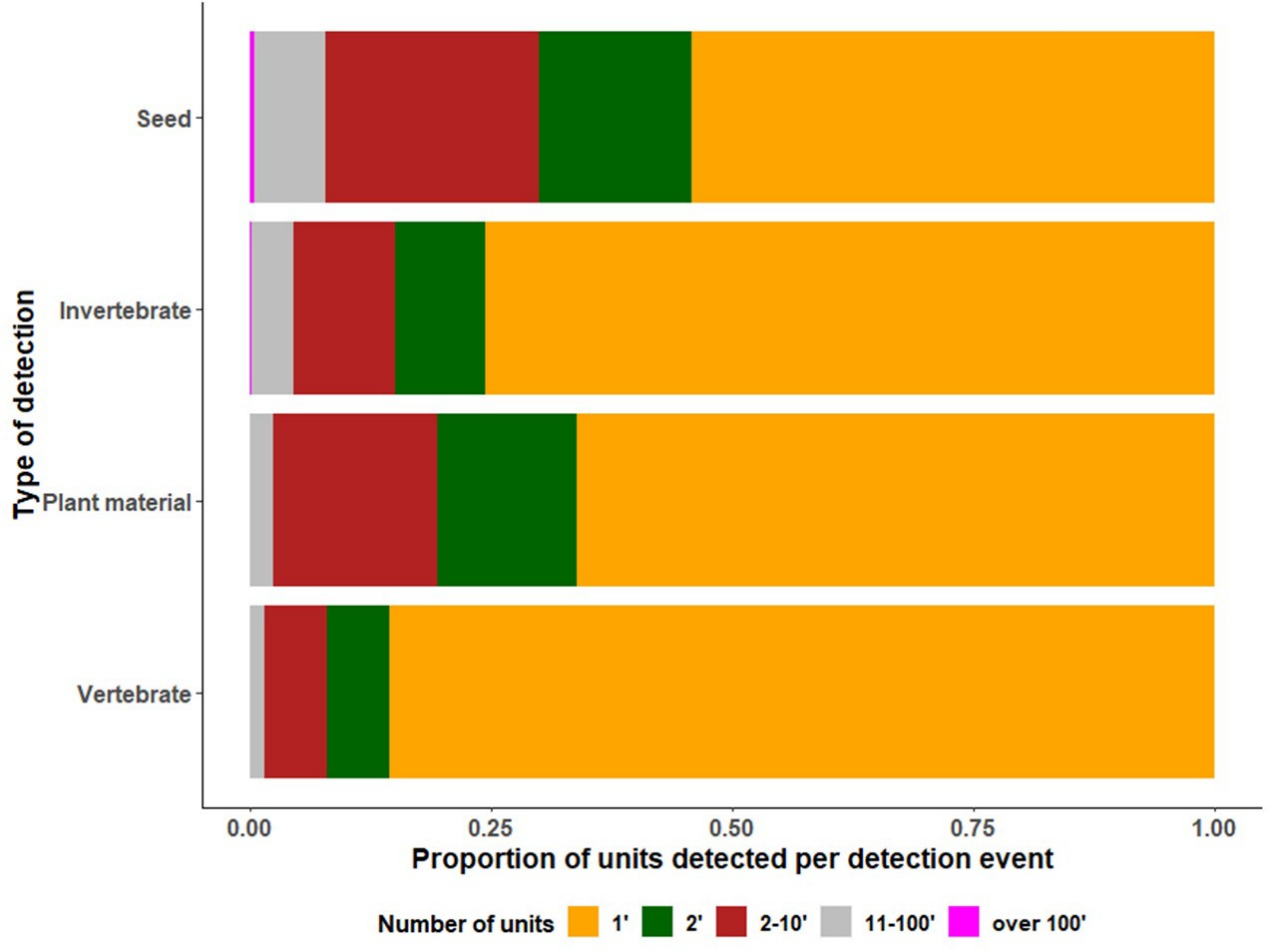
Distribution of detections by type of biosecurity risk material inspected and the number of units detected per inspection for Barrow Island from 2009 to 2015.

**Fig 4:**
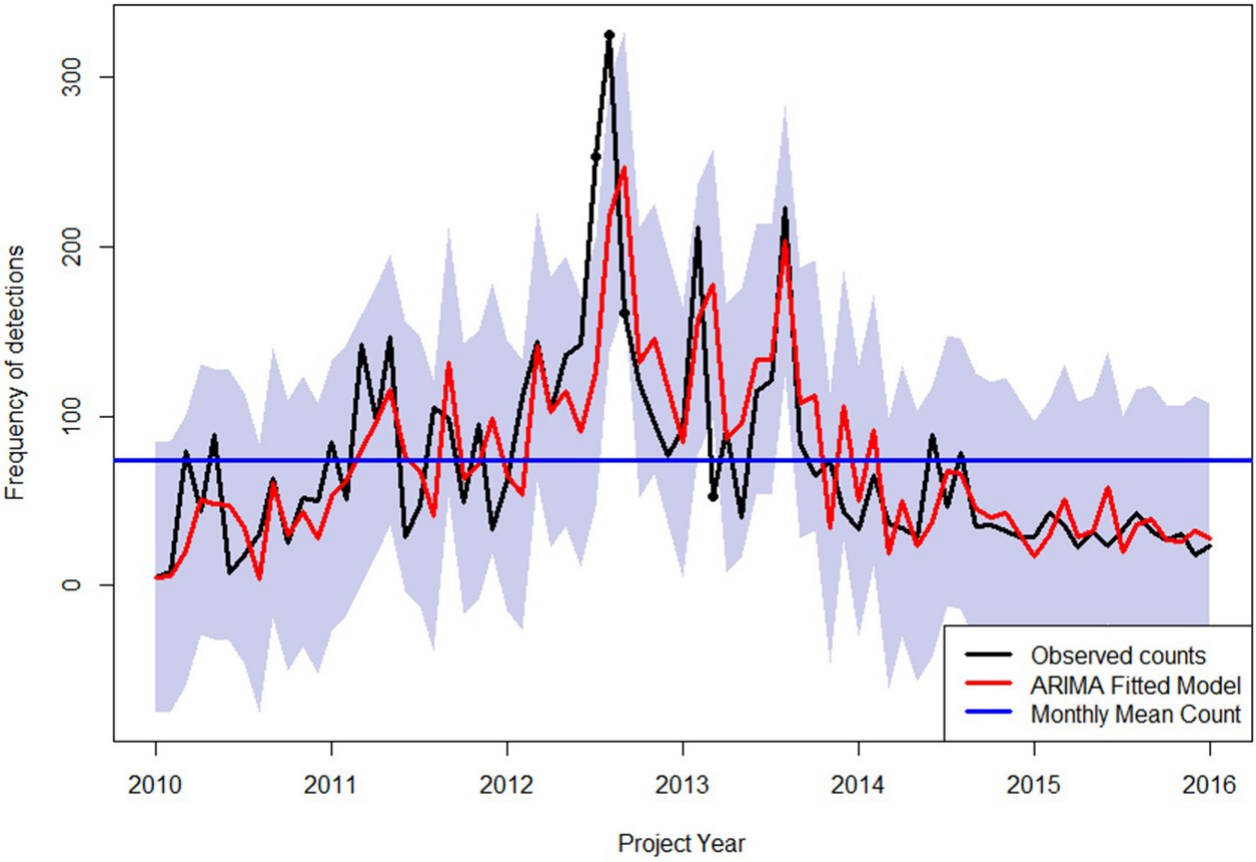
Time series model for total border detection counts between January 2010 and September 2015 for Barrow Island.

Single unit detections comprised 69.1% of the border detections, while 11.4% were two-unit detections. The remaining 19% of detections, ranged between three and 100 units per inspection and only 0.2% ranged from over 100 to a maximum of 1000 units (Fig 3). Since the detections were recorded between 2009 and 2015, temporal autocorrelation between the measurements was intrinsic, recognizing annual, monthly, and seasonal trends. The resulting time series shows a general linear decline in detection counts, with significant spikes at specific points during the inspection period (Fig 4). These detection events were anomalous and mainly comprised of seeds.

#### Cluster analysis of detections

**Fig 5:**
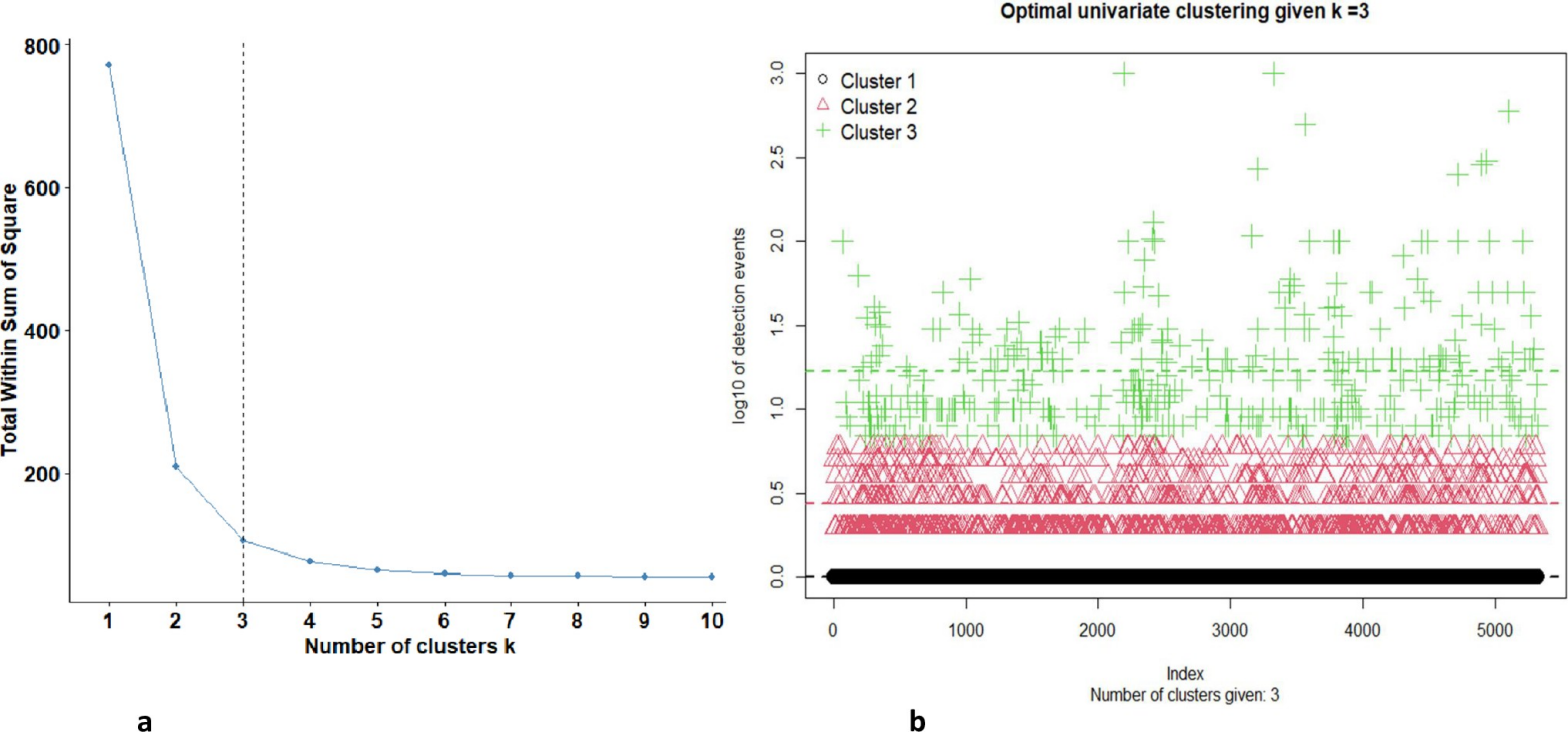
Determining the optimal number of clusters and showing the distribution of the data. a. Scree plot for optimal number of clusters using univariate clustering using ckmeans algorithm for log-transformed data using detection data for Barrow Island from 2009 to 2015. b. Univariate clustering using ckmeans algorithm for log10- transformed data using data for Barrow Island from 2009 to 2015.

Three clusters were identified as optimal for the transformed detection counts using Ckmeans.1d.dp algorithm (Fig 5A). These were from single organism detections, 2 to 6-unit count detections and remaining 7 to 1000 units per detection (Table 3). Single units’ detections accounted for the bulk of the detections (69.4%). The distribution of the 3 clusters is given as a scatter plot (Fig 5B).

**Table 3:** Summary statistics for univariate clustering using ckmeans algorithm for log-transformed data for Barrow Island from 2009 to 2015.

Cluster	Size	Proportion	Mean	std dev	min	median	max	variance
1	3697	69.4	0.0	0	0	0	0	0
2	1185	22.3	0.1185	0.441	0.163	0.301	0.778	0.0264
3	443	8.3	1.23	0.362	0.845	1.15	3	0.131
**Total**	**5325**	**100.0**	**4.030**	**28.164**	**1**	**1**	**1000**	**793.2207**

#### Cluster analysis of detections and covariates

**Fig 6:**
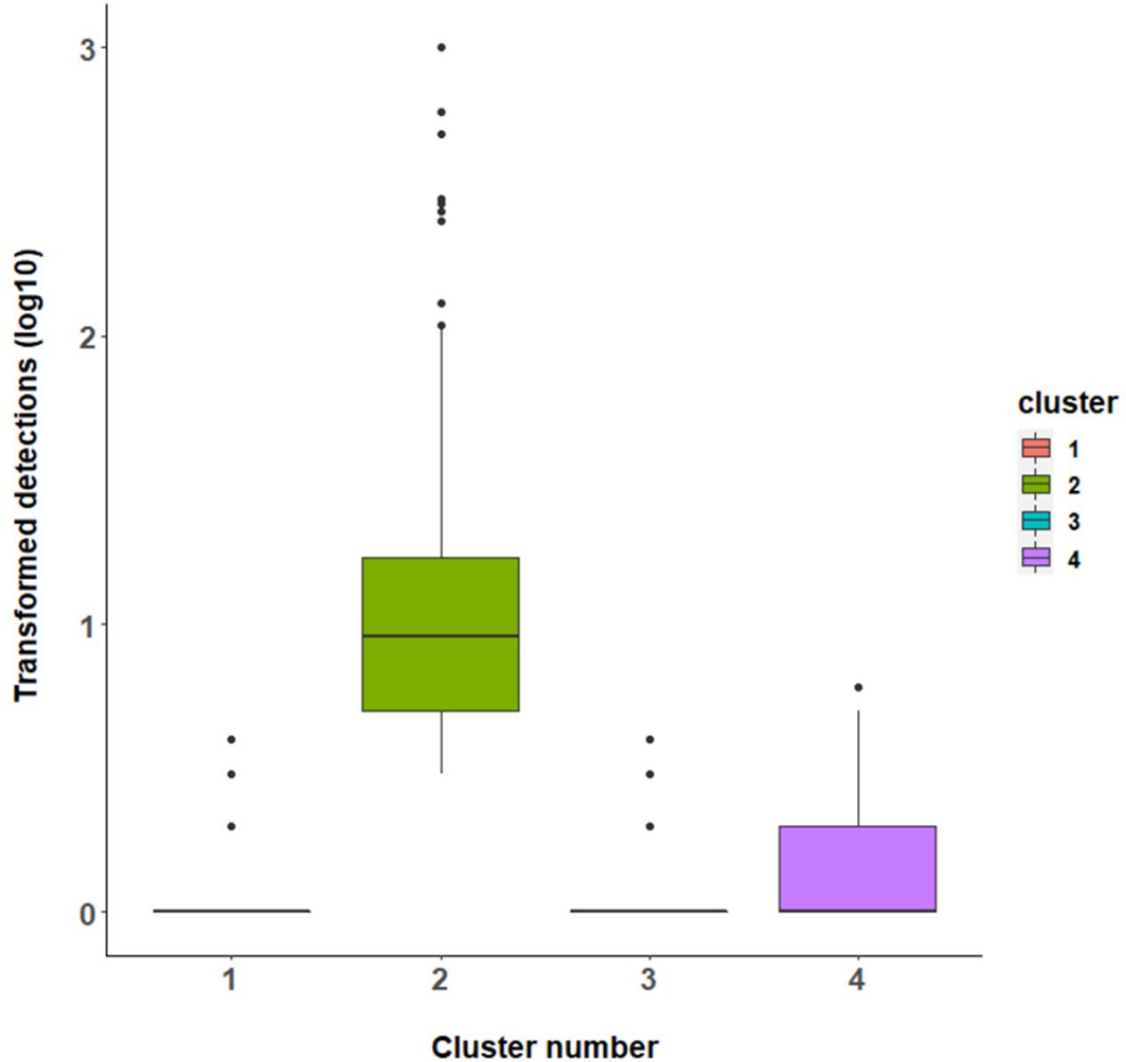
Cluster profile using a boxplot for log10 transformed detection frequency for the four-component cluster model utilizing both numerical and categorical variables for data from Barrow Island from 2009 to 2015.

Four clusters were identified when the following covariates were incorporated into the model using the **clustMixType** package in R: physical location, type of detection, phase, and season. The initial cluster profile of the 4 clusters is shown in Fig 6.

The clusters associated with covariates phase and season had the greatest representation in the detections (Table 4). The parameter lambda (λ = 0.2337) is a measure of the interplay of the different data types for distance calculation during clustering, where small values of λ emphasize numeric variables while larger values show an increased influence of the categorical variables.

**Table 4:** Summary statistics of clustering using the response variable only (counts) for Barrow Island from 2009 to 2015.

Cluster	Size	prop	Mean	std dev	min	median	max	variance
1	904	17.0	1.329	0.662	1	1	4	0.438
2	724	13.6	21.452	74.070	3	9	1000	5486
3	2189	41.1	1.149	0.517	1	1	4	0.268
4	1508	28.3	1.468	0.822	1	1	6	0.675
**Total**	**5325**	**100.0**	**4.030**	**28.164**	**1**	**1**	**1000**	**793.2207**

The clusters were primarily distinguished by season, with no detections in autumn and summer for cluster 1, and no detections in spring for cluster 3. Most of the detections were found in the major construction phase (Table 5). Invertebrates dominated the type of detection for all clusters, constituting 62% of the counts. Cluster 3 is highly associated with WAPET Landing (0.633) and cluster 4 with MOF (0.696) (**See S1 Appendix**).

**Table 5:** Clustering using both numeric and categorical variables using data from Barrow Island between 2009 and 2015.

k-prototype	Cluster size	Mean counts	Detection type				Season
Cluster			Invertebrates	Plant	Seed	Vertebrate	Season
1	904	1.3	0.468	0.124	0.372	0.037	Spring
2	724	21.5	0.503	0.077	0.409	0.011	Winter
3	2189	1.2	0.618	0.093	0.262	0.027	Summer
4	1508	1.45	0.789	0.065	0.123	0.025	Autumn

NB. All clusters were associated with major construction phase. Clusters 1, 2 and 3 were identified with physical location WAPET Landing while cluster 4 with MOF. (**See S1 Appendix**).

**Fig 7:**
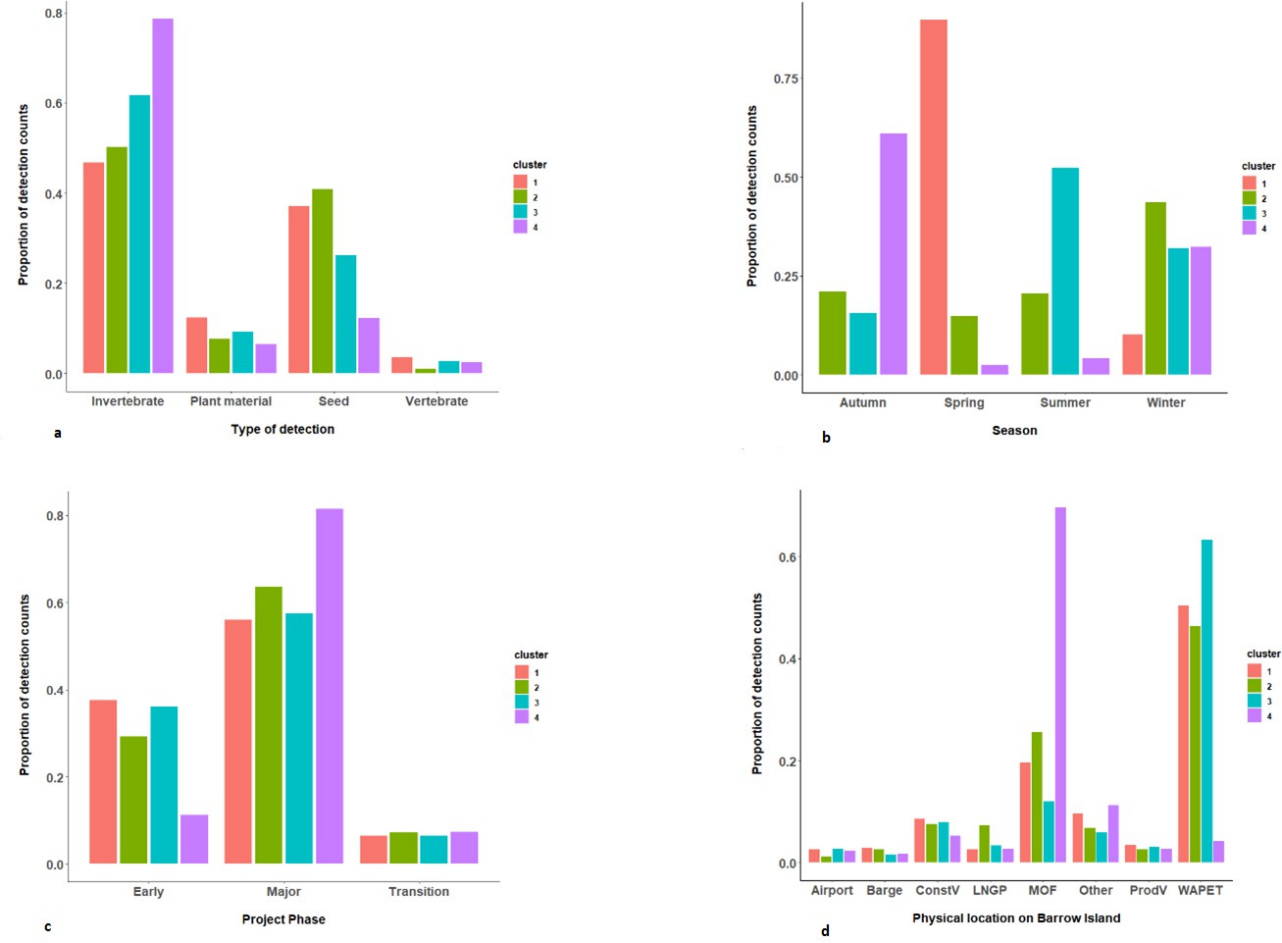
a. Cluster profile by type of detection for the four-component cluster model using data from Barrow Island from 2009 to 2015. b. Cluster profile by seasons for the four-component cluster model using data from Barrow Island from 2009 to 2015. c. Cluster profiles by project phase of construction for the four-component cluster model using data from Barrow Island from 2009 to 2015. d. Cluster profile by physical location of detection event for the four-component cluster model using data from Barrow Island from 2009 to 2015.

The clustering profiles across the different variables showed variations in the distribution of the detection counts within each of the four clusters and by each of the covariates (Fig 7A–7D).

### Cluster performance evaluation

The full cluster model (including both numeric and categorical variables) was compared against the models using numeric variables (k-means) only (Table 6) and using categorical variables (k-mode) (Table 7) using the Rand index [69] as computed in the packages **klaR** [70] and **clusteval** in R [71]. The **Rand index** has a value between 0 and 1, with 0 indicating that the two data clusterings do not agree on any pair of points and 1 indicating that the data clusterings are exactly the same. The rand indices of 0.542 (k-means) and 0.6394 (k-modes) were not high, although the k-modes index was higher and better correlated to both the numerical and categorical variable model. The k-means univariate model accounted for 90.2% of the total variation in the data.

**Table 6:** Summary statistics using numerical variable only for detection data from Barrow Island between 2009 and 2015.

Using numeric variable only	Cluster size	Percentage	Log(detection+1)	Within cluster sum of squares
Cluster1	340	6.4	1.1368	10.592
Cluster2	4304	80.8	0.0424	47.248
Cluster3	616	11.6	0.6043	9.166
Cluster4	65	1.2	1.9085	8.680

**Table 7:** Summary statistics using categorical variables only for detection data from Barrow Island between 2009 and 2015.

Cluster	Cluster size	Percentage	*Within cluster	Detection type	Season	Phase	Physical location
Cluster1	2350	44.1	3335	Invertebrates	Winter	Major	WAPET
Cluster2	1039	19.5	1213	Invertebrates	Summer	Major	WAPET
Cluster3	799	15.0	811	Seed	Summer	Early	WAPET
Cluster4	1137	21.4	959	Invertebrates	Autumn	Major	MOF

N.B. *Within cluster simple-matching distance.

**Fig 8:**
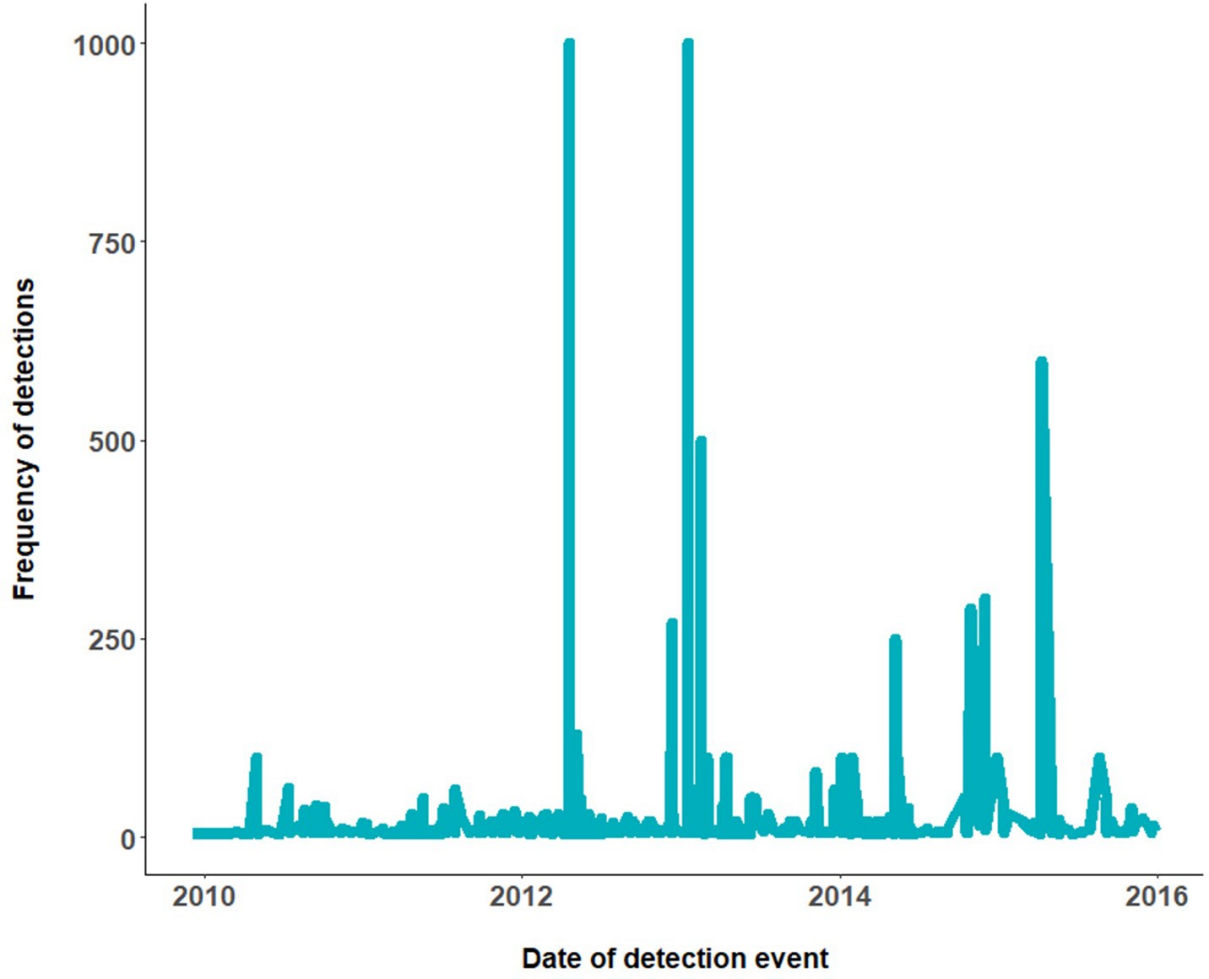
Time series model for biosecurity border detection counts between January 2010 and September 2015 for cluster 2 using data from Barrow Island. N.B. The majority of the detections were single unit detections, with extreme counts of 1000 counts also observed.

Of note was cluster 2 (Table 5) which included the whole range of the detection counts from 1 to 1000 (Fig 8). This cluster represents on a smaller scale the characteristics of biosecurity data commonly encountered. This specific cluster 2 consisting of 724 units, was further analyzed to ascertain whether any extra information would be obtained to better explain the results (Table 8).

**Table 8:** Summary descriptive statistics for cluster 2 for detection data from Barrow Island between 2009 and 2015.

	Min	1^st^ Quartile	Median	Mean	3^rd^ Quartile	Max
Original counts	1	1	1	4.03	2	1000
Log transformed data	0.4771	0.6990	0.9542	1.0025	1.2304	3.00

This new subpopulation of data (cluster 2) identified four clusters that were exclusively from the major construction phase of the project (Table 9). These were characterized by clusters 1 and 2 in which seed were predominantly detected at the LNG Plant and Material Offloading Facility (MOF) respectively. Conversely, clusters 3 and 4 were dominated by invertebrates detected at the WAPET Landing. Cluster 4 did not have any plant material and vertebrates associated with it (**See S2 Appendix**).

**Table 9:** Clustering using both numeric and categorical variables for detection data from Barrow Island between 2009 and 2015.

k-prototype	Cluster size	Mean counts	Detection type				Season	Physical location
			Invertebrates	Plant	Seed	Vertebrate		
Cluster1	101	1.1664	0.099	0.109	0.772	0.020	Winter	LNGPlant
Cluster2	163	0.9806	0.264	0.086	0.632	0.018	Summer	MOF
Cluster3	319	0.7337	0.605	0.097	0.288	0.009	Winter	WAPET
Cluster4	141	1.519	0.837	0000	0.163	0000	Autumn	WAPET

## Discussion

Mixed-data clustering is used to analyse data measured on different scales where the analysis approach is integrative and can identify patterns which are not apparent from univariate analysis of the data [62]. It is also useful for complex high-dimensional data in identifying underlying patterns. Based on our analyses of exemplar biosecurity data collected over a six-year period, we report here that a clustering approach to analysis is useful in interpreting complex patterns in multivariate biosecurity data. Our study indicates that in the Barrow Island (Australia) example, biosecurity border surveillance data for terrestrial non-indigenous species are characterized by homogenous subgroups within a heterogenous population. These data characteristics are analogous to many biosecurity systems globally. Worldwide, the assemblages of organisms transported depends upon the pathway, commodity and suitable environmental conditions, or a combination of these factors. As such, biosecurity data are commonly characterized by presence/absence data (binomial distribution) and clumping and extreme events, which can be characterized by a mixture of distribution models including Poisson, negative binomial distributions, and variants of these such as zero-inflated and hurdle models. Statistical methods used to analyze biosecurity data will be dictated by these distributions.

Determining the most influential variables in biosecurity data is necessary to implement effective biosecurity measures and hence reduce the potential for incursions. In this case study, 1) invertebrates and seeds were demonstrated to be the most abundant NIS detected (contributing 62% and 26% respectively), 2) the highest number of detections were at the WAPET Landing and Material Offloading Facility, which were the primary receival points of cargo at BWI, and 3) most of the detections were during the major construction phase due to the peak in construction activities and human movement. Invertebrates were the most commonly intercepted, as the majority of them are hitchhiker pests attaching themselves to exposed surfaces of ships. A hitchhiker pest is a pest organisms that is moved from one place to another (directly/indirectly) by its opportunistic association with a commodity or item where there is no biological host relationship on/in the conveyance (airplane, maritime vessel, shipping container) used for transport [72].

The ckmeans algorithm identified a three-cluster model as the best model fit based on numerical data. The univariate three-cluster log-normal model provided the best insight into the distribution of the data by dividing data according to their distinct characteristics. Firstly, the model identified point mass detection of single units and the existence of extreme values accounting for the top 2% of the data. Given that biosecurity data often contain extreme values, the log-normal cluster model can be a useful tool for biosecurity management as the transformation allows analysis of all the data collected.

By including categorical covariates in the model, model precision improved by allowing more information to be available to describe the clusters. This was evident by the increase in the number of clusters from three to four. Approximately 90% of the variation in the data was explained by increasing the number of clusters to four using the univariate k-prototype algorithm, a slight improvement from 88.4% with three clusters. This shows inclusion of covariates is important for driving cluster generation.

The four clusters identified in the analysis were mainly distinguished by one of the four seasons and by location on the island, reflecting seasonal and location variations in the data. For example, cluster 1 did not record any detections in summer and autumn, whilst cluster 3 had no detections in spring. In addition, some locations on the island were prominent in the clustering (WAPET Landing and the Material Offloading Facility) because these had the highest number of detections as they were the primary inspection points before the cargo was distributed to various locations on the island. This result supports experts estimates on the relative importance of entry points for incursions on Barrow Island [47, 73]. As such, including categorical covariates in cluster analyses are critical in defining clusters, with specific categorical factors having more weight than others. It is worth noting that the clustering of cluster 2 primarily identified data from the major construction phase, which was the height of construction activity, characterized by increased levels of movement of freight and hence more NIS detection events.

The clustering was able to identify the specific pathways associated with specific cargo that were prominent during the different phases of the project. Introductory pathways associated with cluster 1 were free of NIS detections, namely Sand and Aggregate, Special and Sensitive Goods, and Crated Goods. Further, no detections were recorded in summer and autumn (October–March) for cluster 1. No detections were observed due to the nature of the goods, the transportation pathways and the biosecurity protocols that were applied to their cargo. For example, in the Sand and Aggregate pathway, the sand was deep-mined and stored and transported in containers. While for Crated Goods pathway, the wood was chemically treated according to the Australian timber preservation standards (AS1604). Finally, the Special and Sensitive Goods pathway goods were manufactured or assembled under clinical or hygienic conditions and were thoroughly inspected before being placed in containers, hence no NIS were detected (https://www.standards.org.au/standards-catalogue/sa-snz/building/tm-012/as--1604-dot-1-2012) [74].

Biosecurity protocols in place at Barrow Island have to date resulted in a substantial proportion of biosecurity border inspections (99%) in which no NIS were detected. From over 600,000 border inspections, there were only 5,380 positive detections of NIS i.e., 1% of the total number of inspections. This reflects stringent preventative biosecurity protocols and the limited number of entry points to the island i.e. the seaports and airport [75].

The k-clustering algorithm is generally robust when it comes to extreme values as demonstrated in our biosecurity data as well as in other studies such as Janßen and Wan [76] and Behzadi, Müller [68]. The border detections were highly skewed due to some "extreme values". These extreme values constituted recordings of between 104 to 1000 detections per inspection and were mainly seeds and invertebrate species. Propagule pressure has been identified as a strong predictor of invasion success especially in plants and invertebrate species [12, 77]. Extreme counts, as well as high frequency of detection, are important for biosecurity management strategies as they increase the likelihood of invasion through sufficient propagule pressure [78]. There is increasing empirical and statistical evidence that propagule pressure in the form of propagule sizes, propagule numbers, and temporal and spatial patterns of propagule arrival are important in biological invasions [79]. Establishment success has been shown to depend on propagule pressure in the range of 10 to 100 individuals tested across a broad range of taxa and life histories, including invertebrates, herbaceous plants and long-lived trees, and terrestrial and aquatic vertebrates [77]. As such, extreme detection counts (500+) reported in this study, specifically for *Typha* seeds (bulrush) and one Hymenopteran species, theoretically have the potential to start an invasion and as such provide information critical to biosecurity management. Consequently, our use of clustering to ascertain detection patterns of NIS at the border is highly relevant. Management policy aimed at preventing invasions should aim to reduce detections to small counts or zeros irrespective of any other aspect of an invasion [77]. It is still under debate as to whether and incursion of over 100 individuals have the potential to increase the success of an incursion event. However, extreme counts still need to be taken under careful biosecurity consideration [52].

Given that the numbers required for sufficient propagule pressure are estimated to be 10 to100 individuals [77], the biosecurity protocols in place on Barrow Island limit propagule pressure as 73% of the detections were either single or two-unit detections. However, the efficacy of border controls cannot be evaluated precisely since the actual propagule pressure (frequency of introductions) is unknown [14, 80]. This is often the case in biosecurity situations globally.

Our study revealed that clustering approach has the advantage of catering for heterogeneity of data where subpopulations exist and the data is measured on different scales i.e. numerical or categorical [62]. Border detections at Barrow Island include heterogeneous subpopulations of distinct types of units (vertebrates, invertebrates, seeds, and plant material) because of the nature of cargo and movement of personnel to the island. The characteristics and traits of these types of units vary in their ability to withstand adverse conditions and survive transit, their associated introductory pathway, and commodity. Visual inspections may not be reliable in detecting small or cryptic pests, with the species intercepted possibly attributed to the inspection method used rather than to the association of NIS with the commodity [14, 15]. Thus, even though the clusters identified in statistical analyses may be fit for purpose, the data itself may have specific limitations since not all the relevant data pertaining to biosecurity inspections were available. Factors that might influence the number of detections include the type and quantities of cargo being inspected, the inspection effort, and the method of inspection. This type of dis-segregation of biosecurity data is a common problem faced by data analysts. Until various disciplines and organizations work together, statisticians must manage the available data in the most parsimonious manner, targeting the most effective models for the data they have available.

Most border interception studies investigate a targeted family/phyla of pest or commodity (e.g. the taxonomic and biogeographic patterns of invasion of ants arriving in Australia between 1986 and 2010 [8], border interceptions of forest insects established in Australia between 2003 and 2016 [81], termite interception at United States ports of entry between 1923–2017 [82]. More recent studies in biosecurity have used clustering techniques for automated crop damage assessment [83] and biosecurity investment strategies [84]. Clustering is one of the most popular research topics in data mining and knowledge discovery [85] and using mixed data type is more beneficial as both types of data i.e. numerical and categorical are used together. However, this study looked holistically at all the biosecurity risk material being intercepted at a biosecurity border longitudinally. The method we used produced comparable results using real-life data instead of simulated data as in some studies. Dinh, Huynh [85] gave a comprehensive suite of variations to clustering analysis which demonstrates the wide application of this analysis technique in other areas of research other than biosecurity. However, this study looked holistically at all the biosecurity risk material being intercepted at a biosecurity border over a time and at physical biosecurity inspection points.

## Conclusion

The study highlighted the explanatory variables that best differentiate the spatiotemporal cluster memberships of biosecurity border detection events from a given set of explanatory variables namely type of detection, season, physical location of the detection event and the phase of the project. Cluster models are increasingly becoming an integral component of ecological and environmental data analyses due to their ability to handle diversified data types/mixed-data type to explain the complexity of natural processes and the data itself. Biosecurity surveillance data globally, is often comprised of data measured on different scales (categorical or numerical) and mixed-type data clustering can provide interpretable cluster descriptions which is useful for strategic management decisions in terms of inspection and detection of non-indigenous species. Hence, clustering can be adopted as a tool for investigating and the source and spread of invasive species. This highlights the need to use appropriate statistical methods to explain complex patterns in data such that the information is more readily interpretable for making management decision. Cluster models can identify where subpopulations are aggregated, for example, due to the biological circumstances (e.g., diverse types of organisms transported in the same commodity), but otherwise, exhibiting distinct biological characteristics and traits. Adequate knowledge of how a biosecurity system works is the first step in determining how best to improve the system, and cluster models can be an effective tool in gaining this understanding and indicates that these models should be used more widely in biosecurity monitoring of non-indigenous species.

Even when stringent biosecurity protocols are implemented throughout the biosecurity continuum, it is an impossible task to completely eliminate cargo of contaminants or biosecurity risk material. However stringent protocols minimize the risk of incursions and reduce establishment of pests and diseases in the target environment. Tried and tested protocols, practical innovations and procedures should be shared and improved on progressively as they are tried under different environments and circumstances.

## Supporting information

S1 Appendix Cluster summary information for the complete data set for mixed-type data analysis. (PDF)

S2 Appendix Summary analysis of Cluster 2 identified in the initial complete data set. (PDF)

S1 Dataset Complete data set. (CSV)

S2 Dataset Data for cluster 2. (CSV)
