# Supplementary material for: Use of mixed-type data clustering algorithm for characterizing temporal and spatial distribution of biosecurity border detections of terrestrial non-indigenous species
Source: PLoS One. 2022 Aug 9;17(8):e0272413. doi: 10.1371/journal.pone.0272413 (PMC9362945; doi:10.1371/journal.pone.0272413)
Supplement: S1 Appendix — (PDF) [file pone.0272413.s001.pdf]

S1 : Appendix

Complete data set for mixed-type data analysis

Summary of each cluster by explanatory variable, note for nominal variables it is the percentage contribution of each factor level in that variable summing to 100% across each cluster.

Response variable: log 10 (detection+1) transformation

Cluster	Minimum	1st Quartile	Median	Mean	3rd Quartile	Maximum
1	0.0000	0.0000	0.0000	0.0871	0.0000	0.6021
2	0.4771	0.6989	0.9542	1.0025	1.2304	3.000
3	0.0000	0.0000	0.0000	0.0372	0.0000	0.6021
4	0.0000	0.0000	0.0000	0.1191	0.3010	0.7782

Cluster 2 contained "high" detection counts, while clusters 1 and 3 had the same maximum (0.6021) but different mean values (0.0872 and 0.0372, respectively).

Response variable: Detection type

Cluster	Invertebrate	Plant material	Seed	Vertebrate	Total
1	0.468	0.124	0.372	0.037	1
2	0.503	0.077	0.409	0.011	1
3	0.618	0.093	0.262	0.027	1
4	0.786	0.065	0.123	0.025	1

Invertebrates constitute a significant proportion of the counts for clusters 3 and 4 while for clusters 1 and 2, invertebrates and seeds contributed equally.

Response variable: Season

Cluster	Autumn	Winter	Spring	Summer	Total
1	0.00	0.103	0.987	0.000	1
2	0.210	0.436	0.148	0.206	1
3	0.156	0.320	0.000	0.524	1
4	0.610	0.324	0.025	0.041	1

No detections were found in autumn and summer for cluster 1, while none were detected in spring for cluster 3.

Response variable: Construction phase of the project

Cluster	Early construction	Major construction	Transition	Total
1	0.375	0.561	0.064	1
2	0.293	0.635	0.072	1
3	0.360	0.575	0.065	1
4	0.113	0.814	0.073	1

Most of the detections were found in the major construction phase for cluster 4 while clusters 1 and 3 exhibited similar distribution.

Response variable: Physical location on Barrow Island

Cluster	Barrow Island Airport	Barge*	Construction Village	LNG Plant	Material Offloading Facility	Other ** (POF/ GTP/ QAP)	Production Village	Western Australia Petroleum Landing
1	0.027	0.029	0.086	0.027	0.196	0.096	0.035	0.504
2	0.012	0.026	0.076	0.073	0.256	0.068	0.026	0.463
3	0.027	0.016	0.079	0.033	0.121	0.059	0.031	0.633
4	0.024	0.017	0.053	0.027	0.696	0.113	0.027	0.043

N.B. Barge* is accommodation vessel. Otherincludes Permanent Operating Facility (POF), Gas Treatment Plant (GTP), Quarantine Approved Premises (QAP)**

Cluster 3 is closely associated with WAPET (0.633) and cluster 4 with MOF (0.696). In cluster1 Barrow Island Airport and LNG Plant had similar detection proportions (0.027) though negligible. Similarly, in cluster 2, Production Village and Barge had similar detection proportion (0.026).
