# Supplementary material for: Use of mixed-type data clustering algorithm for characterizing temporal and spatial distribution of biosecurity border detections of terrestrial non-indigenous species
Source: PLoS One. 2022 Aug 9;17(8):e0272413. doi: 10.1371/journal.pone.0272413 (PMC9362945; doi:10.1371/journal.pone.0272413)
Supplement: S2 Appendix — (PDF) [file pone.0272413.s002.pdf]

S2: Appendix

Summary information for the analysis of Cluster 2 identified in the initial complete data set

Response variable: log10 (detect+1); n=724

Minimum	1st Quartile	Median	Mean	3rd Quartile	Maximum
0.4771	0.6990	0.9542	1.0025	1.2304	3.0000

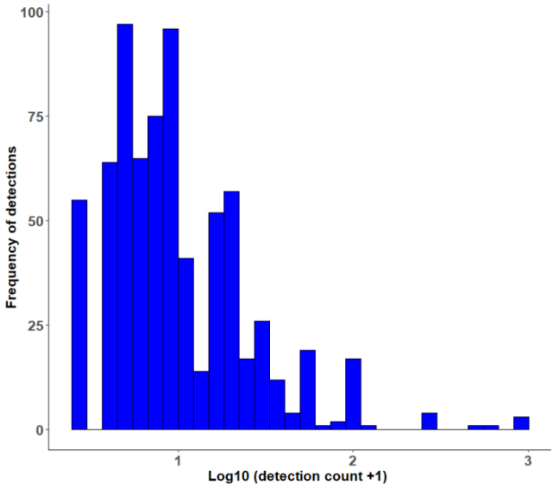

Fig 1: Data distribution for cluster 2 (n=724).

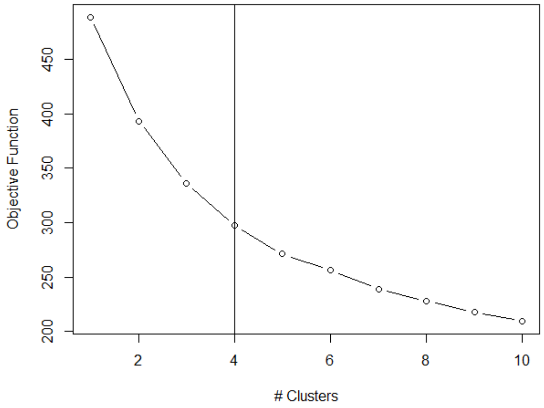

Fig 2: Scree plot for identifying optimal cluster size for cluster 2

Clustering using covariates only

Numeric predictors: 1
Categorical predictors: 4
Lambda: 0.2613846

Number of Clusters: 4
Cluster sizes: 101 163 319 141
Within cluster error: 38.307;86.413; 143.163; 81.677

Cluster prototypes:

Cluster	Log10(detect+1)	Detection.Type	Season	Phase of construction	Physical location
1	1.166	Seed	Winter	Major	LNGP
2	0.981	Seed	Summer	Major	MOF
3	0.734	Invertebrate	Winter	Major	WAPET
4	1.519	Invertebrate	Autumn	Major	WAPET

Response variable: log 10 (detect+1)

Cluster	Minimum	1st Quartile	Median	Mean	3rd Quartile	Maximum
1	0.477	1.000	1.114	1.166	1.301	2.000
2	0.602	0.778	0.954	0.981	1.041	1.914
3	0.477	0.602	0.699	0.734	0.845	1.230
4	1.000	1.279	1.398	1.519	1.699	3.000

Response variable: Detection type

Cluster	Invertebrate	Plant material	Seed	Vertebrate	Total
1	0.099	0.109	0.772	0.020	1
2	0.264	0.086	0.632	0.018	1
3	0.605	0.097	0.288	0.009	1
4	0.837	0.000	0.163	0.000	1

Response variable: Season

Cluster	Autumn	Winter	Spring	Summer	Total
1	0.030	0.851	0.119	0.000	1
2	0.141	0.104	0.221	0.534	1
3	0.191	0.580	0.129	0.100	1
4	0.461	0.199	0.128	0.213	1

Response variable: Construction phase of the project

Cluster	Early construction	Major construction	Transition	Total
1	0.109	0.871	0.020	1
2	0.417	0.466	0.117	1
3	0.317	0.624	0.060	1
4	0.227	0.688	0.085	1

Response variable: Physical location on Barrow Island

Cluster	Barrow Island Airport	Barge*	Construction Village	LNG Plant	Material Offloading Facility	Other ** (POF/ GTP/ QAP)	Production Village	Western Australia Petroleum Landing
1	0.000	0.010	0.030	0.426	0.109	0.089	0.030	0.307
2	0.037	0.018	0.018	0.012	0.552	0.067	0.006	0.288
3	0.006	0.028	0.082	0.016	0.172	0.060	0.034	0.602
4	0.007	0.043	0.163	0.021	0.206	0.071	0.028	0.461

N.B Barge* is accommodation vessel. Otherincludes Permanent Operating Facility (POF), Gas Treatment Plant (GTP), Quarantine Approved Premises (QAP)**

Clustering using numerical data only

K-means clustering with 4 clusters of sizes 205, 9, 452, 58

Cluster means:

Cluster	Cluster size	Log10 (detect+1)
1	205	1.245
2	9	2.693
3	452	0.760
4	58	1.773

Within cluster sum of squares by cluster:
[1] 3.7193882 0.5467195 12.4459140 1.8280527
(between_SS / total_SS = 84.2 %)

1. Clustering using categorical data only

Cluster	Cluster size	Detection.Type `	Season	Phase construction	Physical location
1	214	Invertebrate	Spring	Early	WAPET
2	269	Seed	Winter	Major	WAPET
3	158	Invertebrate	Summer	Major	MOF
4	83	Invertebrate	Autumn	Major	WAPET

2. Clustering using both numerical and categorical data

Cluster	Cluster size	Log10(detect+1)	Detection.Type	Season	Construction Phase	Physical location
1	183	0.978	Invertebrate	Autumn	Major	WAPET
2	217	1.001	Invertebrate	Winter	Major	MOF
3	243	0.758	Seed	Winter	Major	WAPET
4	81	1.771	Seed	Winter	Major	WAPET
